# Long-term efficacy and safety of rituximab in IgG4-related disease: Data from a French nationwide study of thirty-three patients

**DOI:** 10.1371/journal.pone.0183844

**Published:** 2017-09-15

**Authors:** Mikael Ebbo, Aurélie Grados, Maxime Samson, Matthieu Groh, Anderson Loundou, Aude Rigolet, Benjamin Terrier, Constance Guillaud, Clarisse Carra-Dallière, Frédéric Renou, Agnieszka Pozdzik, Pierre Labauge, Sylvain Palat, Jean-Marie Berthelot, Jean-Loup Pennaforte, Alain Wynckel, Céline Lebas, Noémie Le Gouellec, Thomas Quémeneur, Karine Dahan, Franck Carbonnel, Gaëlle Leroux, Antoinette Perlat, Alexis Mathian, Patrice Cacoub, Eric Hachulla, Nathalie Costedoat-Chalumeau, Jean-Robert Harlé, Nicolas Schleinitz

**Affiliations:** 1 Department of Internal Medicine, Hôpital de la Timone, AP-HM, Aix-Marseille Université, Marseille, France; 2 Department of Internal Medicine and Clinical Immunology, Dijon University Hospital, Dijon, France; 3 Department of Internal Medicine, Hôpital Cochin, AP-HP, Centre National de Référence Maladies Systémiques et Auto-immunes Rares, Université René-Descartes Paris V, Sorbonne Paris Cité, Paris, France; 4 Unité d’Aide Méthodologique, Aix-Marseille Université, AP-HM, Marseille, France; 5 Department of Internal Medicine and Clinical Immunology, AP-HP, Hôpital La Pitié-Salpêtrière; DHUI2B, Université Pierre et Marie Curie Paris VI, Paris, France; 6 Department of Internal Medicine, Hôpital Henri Mondor, AP-HP, Créteil, France; 7 Department of Neurology, CHRU de Montpellier, Montpellier, France; 8 Department of Internal Medicine, CHU La Réunion site Félix Guyon, Saint-Denis, La Réunion, France; 9 Department of Nephrology, Erasme Hospital, Cliniques Universitaires de Bruxelles, Bruxelles, Belgium; 10 Department of Internal Medicine, CHU Limoges, Limoges, France; 11 Department of Rheumatology, CHU Nantes, Nantes, France; 12 Department of Internal Medicine, CHU de Reims, Reims, France; 13 Department of Nephrology, CHU de Reims, Reims, France; 14 Department of Nephrology, CHRU de Lille, Lille, France; 15 Department of Nephrology and Internal Medicine, CH Valenciennes, Valenciennes, France; 16 Department of Nephrology, Hôpital Tenon, AP-HP, Paris, France; 17 Department of Gastro-enterology, CHU Bicêtre, Le Kremlin Bicêtre, France; 18 Department of Internal Medicine, Hôpital Sud, CHU de Rennes, Rennes, France; 19 Department of Internal Medicine, Hôpital Pitié-Salpêtrière, AP-HP, Centre National de Référence Maladies Systémiques et Auto-immunes Rares, Université Pierre et Marie Curie Paris VI, Paris, France; 20 National Referral Centre for Auto-immune and Systemic Diseases, Department of Internal Medicine, Huriez Hospital, Université de Lille, Lille, France; Klinikum rechts der Isar der Technischen Universitat Munchen, GERMANY

## Abstract

**Objectives:**

To assess efficacy and safety of rituximab (RTX) as induction therapy, maintenance of remission and treatment of relapses in a cohort of IgG4-related disease (IgG4-RD) patients.

**Methods:**

Nationwide retrospective multicenter study of IgG4-RD patients treated with at least one course of RTX. Clinical, biological and radiological response, relapse rate and drug tolerance were analyzed. Kaplan-Meier curves were plotted and risk factors for relapse studied with a Cox regression model.

**Results:**

Among 156 IgG4-RD patients included in the French database, 33 received rituximab. Clinical response was noted in 29/31 (93.5%) symptomatic patients. Glucocorticoids withdrawal was achieved in 17 (51.5%) patients. During a mean follow-up of 24.8 ±21 months, 13/31 (41.9%) responder patients relapsed after a mean delay of 19 ±11 months after RTX. Active disease, as defined by an IgG4-RD Responder Index >9 before RTX, was significantly associated with relapse (HR = 3.68, 95% CI: 1.1, 12.6) (*P* = 0.04), whereas maintenance therapy with systematic (i.e. before occurrence of a relapse) RTX retreatment was associated with longer relapse-free survival (41 versus 21 months; *P* = 0.02). Eight severe infections occurred in 4 patients during follow-up (severe infections rate of 12.1/100 patient-years) and hypogammaglobulinemia ≤5 g/l in 3 patients.

**Conclusion:**

RTX is effective for both induction therapy and treatment of relapses in IgG4-RD, but relapses are frequent after B-cell reconstitution. Maintenance therapy with systematic RTX infusions is associated with longer relapse-free survival and might represent a novel treatment strategy. Yet, the high rate of infections and the temporary effect of RTX might be hindrances to such strategy.

## Introduction

IgG4-related disease (IgG4-RD) is a recently individualized systemic fibro-inflammatory disorder with characteristic histopathological lesions within involved organs, heterogeneous clinical presentation and unknown pathophysiology [1].

Dramatic response to glucocorticoids (GC) is usual [2–4] and has been retained as an optional criterion in some organ-specific diagnostic criteria of the disease [5,6]. However, 30 to 60% of patients relapse during the tapering or after withdrawal of GC [4,7–9]. In addition, long-term GC use is associated with frequent side effects, namely diabetes mellitus, infections, or osteoporotic fractures in these usually middle aged patients [4,9,10]. Hence, the evaluation of steroid-sparing agents is warranted and represents a major challenge in the management of patients with IgG4-RD.

Minimal data has been published on the use of conventional immunosuppressive agents (e.g. azathioprine, mycophenolate mofetil, or methotrexate) for the treatment of IgG4-RD patients [4,8,9,11,12]. Promising results have been reported with rituximab (RTX, an anti-CD20 monoclonal B-cell depleting antibody) in preliminary case reports [13–16], case series [17–19] and in an open, non-controlled trial [20]. Yet, data of long-term follow-up of patients treated with RTX in daily care are scarce [18,21]. Herein, we report on the use of RTX in patients with IgG4-RD enrolled in a nationwide French multicenter cohort.

## Patients and methods

### Study design, inclusion criteria and outcome assessment

Between 2009 and 2016, a total of 156 patients fulfilling the Comprehensive Diagnostic Criteria for IgG4-RD [22] and/or specific organ diagnostic criteria for type 1 autoimmune pancreatitis [5], IgG4-related sclerosing cholangitis [6], IgG4-related sialadenitis/dacryoadenitis [23], and IgG4-related nephritis [24,25] were included in the French multicenter database for IgG4-RD. IgG4-RD patients treated with RTX for this indication and with at least 3 months of follow-up available after treatment onset were included in the study. Data regarding clinical manifestations, radiology and laboratory findings at diagnosis and during follow-up were retrospectively collected from each center.

Disease activity before treatment was evaluated by the IgG4-RD Responder Index (IgG4-RD RI) [26], excluding the measurement of serum IgG4 levels. Outcome after RTX treatment was assessed by the evaluation of clinical (improvement or resolution of symptomatic organ involvement), radiological (improvement or resolution of radiological abnormalities in conventional and/or metabolic imaging studies) and biological response (improvement or normalization of initially abnormal serological markers, including standard laboratory results and more characteristic serological markers such as serum IgG4 levels, IgE concentrations, eosinophilia, and complement levels). Relapse was defined by any new clinical, radiological, or biological manifestation specifically related to IgG4-RD. Isolated high serum IgG4 level was not considered as a relapse per se.

To assess tolerance, all clinical events occurring during and after RTX treatment were collected.

This study complies with the current French Legislation, which mentions that retrospective observational studies that do not change the routine management of patients do not need to be declared or submitted to the opinion of a research ethics board (Loi Jardé relating to research involving humans, decree n° 2016–1537, 16 November 2016). The French multicenter database for IgG4-RD was approved by French authorities (Comité Consultatif sur le Traitement de l’Information en matière de Recherche dans le domaine de la Santé (CCTIRS) and Commission Nationale de l’Informatique et des Libertés (CNIL)). All data were fully anonymized before we accessed them.

### Statistical analysis

Statistical analysis was performed using IBM SPSS Statistics for Windows, Version 20.0 (IBM SPSS Inc., Chicago, IL, United States [US]). Continuous variables were expressed as mean ±SD or as median with interquartile range (IQR) or range (min–max), and categorical variables were reported as count and percentages. Comparisons of mean values between two groups were performed using Mann-Whitney U or student t-tests. Comparisons of percentages were performed using Chi-Square or Fisher’s exact tests, as appropriate. Time-to-relapse endpoint was estimated by the Kaplan–Meier method and compared using the log-rank test. Univariate and multivariate Cox proportional hazard regression models were used to estimate the hazard ratio (HR). Hazards ratio were expressed with 95% confidence intervals (CI 95%). For all tests, a *P*-value <0.05 was considered significant.

## Results

### Patients’ characteristics

Among patients included in the French multicenter database for IgG4-RD, 43/156 (27.6%) were treated with at least one RTX infusion and 33 were analyzed in the study (5 patients were excluded due to insufficient data for IgG4-RD diagnosis, 4 due to short (i.e. <3 months) follow-up, and a single patient was treated with RTX for another indication (S1 Fig).

Patients’ characteristics are presented in Table 1. The male/female ratio was 2.7/1 and mean age at RTX was 57.7 ±12.7 years (range: 31–84). Thirty patients had biopsy-proven disease: 20 patients (60.6%) presented with definite diagnosis of IgG4-RD, and 10 (30.3%) with probable diagnosis [22]. Three patients (9.1%) presented with possible diagnosis.

**Table 1:** Demographic, pathological, clinical and biological baseline characteristics of 33 patients with IgG4-RD treated with RTX.

Parameters	Values
Mean age at RTX treatment, years (±SD)	57.7 (±12.7)
Male gender, n (%)	24 (72.7)
Mean duration of IgG4-RD before RTX, months [range]	41 [4–180]
Biopsy proven disease, n (%)	30 (90.1)
**Comprehensive Diagnostic Criteria**	**n (%)**
Definite	20 (60.6)
Probable	10 (30.3)
Possible	3 (9.1)
Number of organs affected, mean [range]	3.2 [1–8]
IgG4-RD Responder Index, mean [range]	10 [2–24]
**Organ site involvement**	**n (%)**
Lymphadenopathy	18 (54.5)
Pancreas	15 (45.5)
Biliary duct	13 (39.4)
Kidney	10 (30.3)
Orbit	8 (24.2)
Salivary gland	8 (24.2)
Retroperitoneal fibrosis	5 (15.2)
Prostate	5 (15.2)
Aorta/arterial involvement	4 (12.1)
Lacrimal gland	4 (12.1)
Other IPT	4 (12.1)
Lung	3 (9.1)
**Laboratory parameters before RTX**	**n (%)**
Elevated serum IgG4 >135 mg/dl	24 (72.7)
Hypergammaglobulinemia >15 g/l	14/29 (48.3)
Eosinophilia >0.5 G/l	7/31 (22.6)
Elevated serum IgE	9/11 (81.8)
C3/C4 or CH50 decrease	4/20 (20)
CRP >10 mg/l	9/29 (31)

CRP, C-reactive protein; IPT, Inflammatory Pseudo-Tumor; RD, related disease; RTX, rituximab; SD, standard deviation.

Mean number of involved organs was 3.2 ±0.7 (range: 1–8) and 19 (57.6%) patients presented with ≥3-affected organs. Involved organs were predominantly lymph nodes (54.5%), pancreas (45.5%), bile ducts (39.4%), kidneys (30.3%), orbits (24.2%), salivary glands (24.2%), and retroperitoneum (15.2%). The disease was limited to one organ or tissue in 10 patients (orbit in seven patients, and aortitis, retroperitoneal fibrosis or cholangitis in one patient each). Mean IgG4-RD RI (excluding serum IgG4 levels) [26] before RTX treatment was 10 ±6.1 (range: 2–24). RTX was used in case of “urgent” localization (as defined in [27]) in 21 (63.6%) patients.

Twenty-four patients (72.7%) presented with elevated (>135 mg/dl) serum IgG4 levels (median: 550 mg/dl (IQR: 325–775)). Hypocomplementemia was found in 4/20 patients (20%) and was systematically associated with kidney involvement. Eosinophilia (>0.5 G/l) or C-reactive protein (CRP) >10 mg/l were found in 22.6% and 31% of patients respectively.

### Previous treatments for IgG4-RD

Treatments received before the first infusion of RTX are listed in Table 2. All patients but 2 had received at least one treatment before the first RTX infusion. The mean number of previous medical treatments was 1.6 ±1 (range: 0–4). Thirty-one patients (93.9%) had undergone previous glucocorticoids (GC) courses, among which 24 (77.4%) were steroid-dependent relapsing during decrease (n = 11) or after withdrawal (n = 13). Response to GC was considered insufficient in six cases (19.4%). Two patients (with IgG4-related tubulointerstitial nephritis and cholangitis, respectively) were considered as steroid non-responders. Thirteen patients (39.4%) had already received steroid-sparing agents (azathioprine (n = 9), methotrexate (MTX) (n = 4), cyclophosphamide (n = 1), or mycophenolate mofetil (MMF) (n = 1)) prior to RTX infusions. Relapses justifying RTX occurred in nine of them (69.2%) under these treatments. Fourteen patients (42.4%) underwent interventional procedures before RTX: urinary or biliary endoscopic derivation in two and four cases, respectively, and surgical treatment in eight cases. Three patients received RTX as first-line treatment either without GC (contra-indication to GC in the context of infectious osteitis and tuberculosis in one case) or in association with GC (RTX was used as a steroid-sparing agent in an obese, diabetic patient with bipolar disorder, and for the treatment of another patient with severe disease-specific pharyngeal involvement).

**Table 2:** Previous treatments received for IgG4-RD prior to RTX in 33 patients with IgG4-RD treated with RTX.

Treatments	
Number of previous medical treatments, mean [range]	1.6 [0–4]
Previous glucocorticoid course(s), n (%)	31 (93.9)
Previous DMARD treatment, n (%)	13 (39.4)
Azathioprine	9 (27.3)
Methotrexate	4 (12.1)
Cyclophosphamide	1 (3)
Mycophenolate mofetil	1 (3)
Other	4 (12.1)
Previous interventional treatment, n (%)	
Urinary derivation	2 (6.1)
Biliary stenting	4 (12.1)
Previous surgical treatment, n (%)	8 (24.2)

DMARD, disease modifying anti-rheumatic drug; RTX, rituximab.

### RTX regimen and concomitant treatments

Intravenous RTX regimens included two doses of 1000 mg separated by 15 days in 25 patients (75.8%), 4 weekly doses of 375 mg/m^2^ in six patients (18.2%) and other regimens in two patients (4 weekly doses of 150 mg/m^2^ or 2 doses of 375 mg/m^2^ separated by 15 days). All patients received premedication with IV methylprednisolone (100 mg), acetaminophen (1000 mg) and dexchlorpheniramine (5 mg). Steroids were associated with RTX in all patients except one.

In four patients (12.1%), disease-modifying anti-rheumatic drugs (DMARDs) were maintained in association with RTX (azathioprine in three patients, MMF in one patient).

### Disease response to RTX

Among 31 clinically symptomatic patients before onset of RTX, 29 (93.5%) presented a clinical response after treatment (Table 3). The response occurred within 1 month of RTX treatment. The two non-responders presented with meningeal inflammatory pseudo-tumor (IPT) and maxillary IPT. No difference was observed in terms of clinical response between patients with definite and probable/possible IgG4-RD diagnosis (clinical response in 94% and 92.3%, respectively).

**Table 3:** Clinical, biological and radiological efficacy of RTX in 33 patients with IgG4-RD.

Outcome	n (%)
**RTX regimen**	
1 g d1-d15	25 (75.8)
375 mg/m^2^/w x4	6 (18.2)
Other	2 (6.1)
**Clinical response**^a^	29/31 (93.5)
**Biological response on serum IgG4**^b^	
Serum IgG4 decrease >50%	16/19 (84.2)
Serum IgG4 normalization (<135 mg/dl)	14/19 (73.7)
**Biological response on non-specific biology**^c^	21/23 (91.3)
**Radiological response** (conventional, CT or MRI)	
Radiological stabilization	5/13 (38.5)
Radiological improvement	4/13 (30.75)
Radiological normalization	4/13 (30.75)
**Metabolic response** (FDG-PET/CT)	
No metabolic response/worsening	1/14 (7.1)
Metabolic stabilization	3/14 (21.4)
Metabolic improvement	4/14 (28.6)
Metabolic normalization	6/14 (42.9)
**Glucocorticoid withdrawal**^d^	17/33 (51.5)
**DMARD treatment**^d^	6/33 (18.2)
**Mean follow-up**, months (±SD)	24.8 (±21.0)
**Relapse**^e^	13/31 (41.9)
Mean time to relapse, months (±SD)	19.0 (±11.5)
**Retreatment with RTX**	17/33 (51.5)
Systematic/maintenance retreatment^f^	12/33 (36.4)
Retreatment for relapse^f^	9/33 (27.2)

^a^ Evaluated in 31 symptomatic patients at RTX treatment.

^b^ In patients with pre-rituximab serum IgG4 elevation and available values of serum IgG4 during follow-up after RTX.

^c^ Non-specific biology: renal, hepatic biological results, C-reactive protein, complement, IgE or other biological abnormalities in relation to the disease.

^d^ At last follow-up.

^e^ Evaluated in 31 responder patients.

^f^ The same patient could have been successively retreated systematically and for relapse.

d, day; FDG-PET/CT, ^18^F-fluoro-2-deoxy-D-glucose positron emission tomography/computed tomography; RTX, rituximab; SD, standard deviation; w, week.

Among 19 patients with baseline elevated serum IgG4 levels, a >50% decrease of serum IgG4 level was observed in 16 (84.2%) with a median delay of 3 months (range: 1–6) after treatment, and normalization in 14 (73.7%) with a median delay of 6 months (range: 1–16) after treatment. Other laboratory tests (either organ-dependent: creatininemia, liver or pancreatic tests, or non-specific: CRP, complement, eosinophilia, or IgE elevation) were abnormal before RTX in 23 patients and their improvement or normalization occurred in 91.3% (n = 21) of cases. Radiological examinations (CT or MRI) were available before and after RTX in 13 cases, and showed stabilization in 5 (38.5%), improvement in 4 (30.7%) and normalization in 4 (30.7%) cases. Evaluation by 18 FDG-PET/CT was available before and after RTX in 14 cases, showing stabilization (unchanged) in 3, improvement (significant decrease of standardized uptake values and/or size of hypermetabolic lesions) in 4, normalization in 6, and worsening in 1 case.

Mean follow-up after first RTX infusion was 24.8 ±21 months (IQR: 12.5–31.5, range: 3–109). GC withdrawal before last visit was achieved in 17 patients (51.5%). The mean dose of GC at last follow-up of patients with GC maintenance therapy was 9.6 ±9.3 mg/d (range: 4–40). Six patients (18.2%) remained under DMARDs at last follow-up (azathioprine in 3 patients, MMF in 2, and MTX in 1) (Table 3).

### Predictive factors of relapse

**Fig 1:**
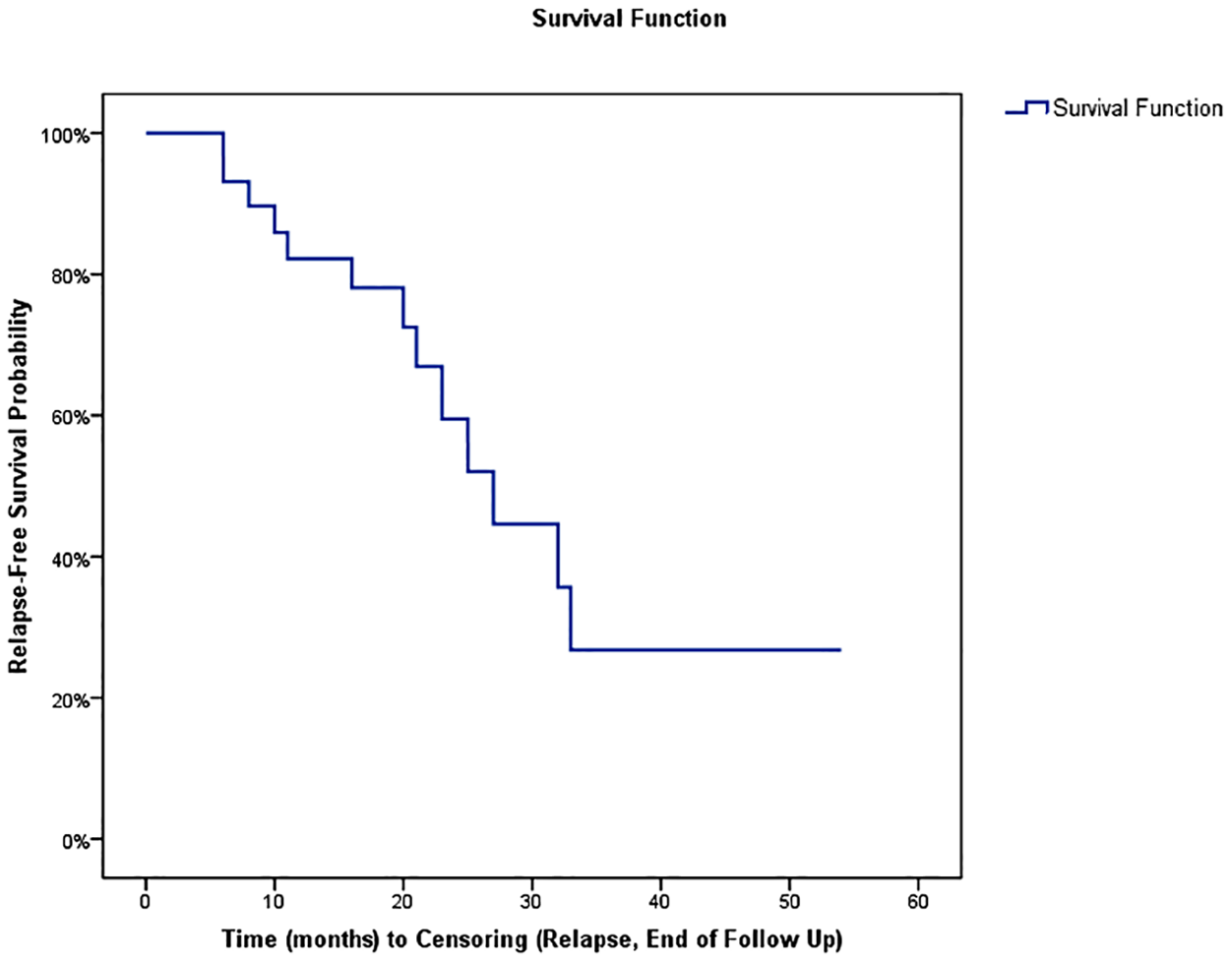
Kaplan-Meier plots of the risk of disease relapse following rituximab in 31 IgG4-RD responding patients.

The relapse rate after onset of treatment with RTX was analyzed in 31 patients considered as ‘responders’ because of a clinical response in 29 clinically symptomatic patients, a biological response in 1 clinically asymptomatic patient with renal involvement, and a radiological response in 1 clinically asymptomatic patient with retroperitoneal fibrosis. Relapse was observed in 13 of 31 responders (41.9%) during follow-up. The mean delay of relapse was 19 ±11 months (median 17, IQR: 10–25, range: 3–54) and the mean relapse-free survival was 30 ±4 months (IQR: 22.6–37.9) (Fig 1). Organs involved during relapses were kidney and pancreas in 4 cases each, lung, salivary glands and bile ducts in 3 cases each, orbit and aorta in 2 cases each, and lachrymal glands, retroperitoneum, prostate, maxillary IPT and skin in 1 case each. There was no difference in the rates of relapses between patients with definite and probable/possible IgG4-RD diagnosis (relapses in 40% and 38.5%, respectively). Five patients relapsed after GC discontinuation, whereas the mean dose of GC was 5.3 mg/d in 8 patients under maintenance therapy with GC at the time of relapse. Among 17 responding patients with initial serum IgG4 decrease (one patient presented a decrease of serum IgG4 after rituximab but no clinical response and was not included in this analysis), 11 (64.7%) presented a persistent decrease or normalization of IgG4 levels after a median follow-up of 17 months (range: 3–33). Yet, 3 of these patients (27.2%) relapsed despite normal (n = 2) or persistent decreased (n = 1) serum IgG4 levels. In 6 patients (35.3%), a new elevation of serum IgG4 levels occurred after RTX treatment with a median delay of 17 months (range: 10–27), and all of them had concomitant clinical and/or radiological relapse.

**Fig 2:**
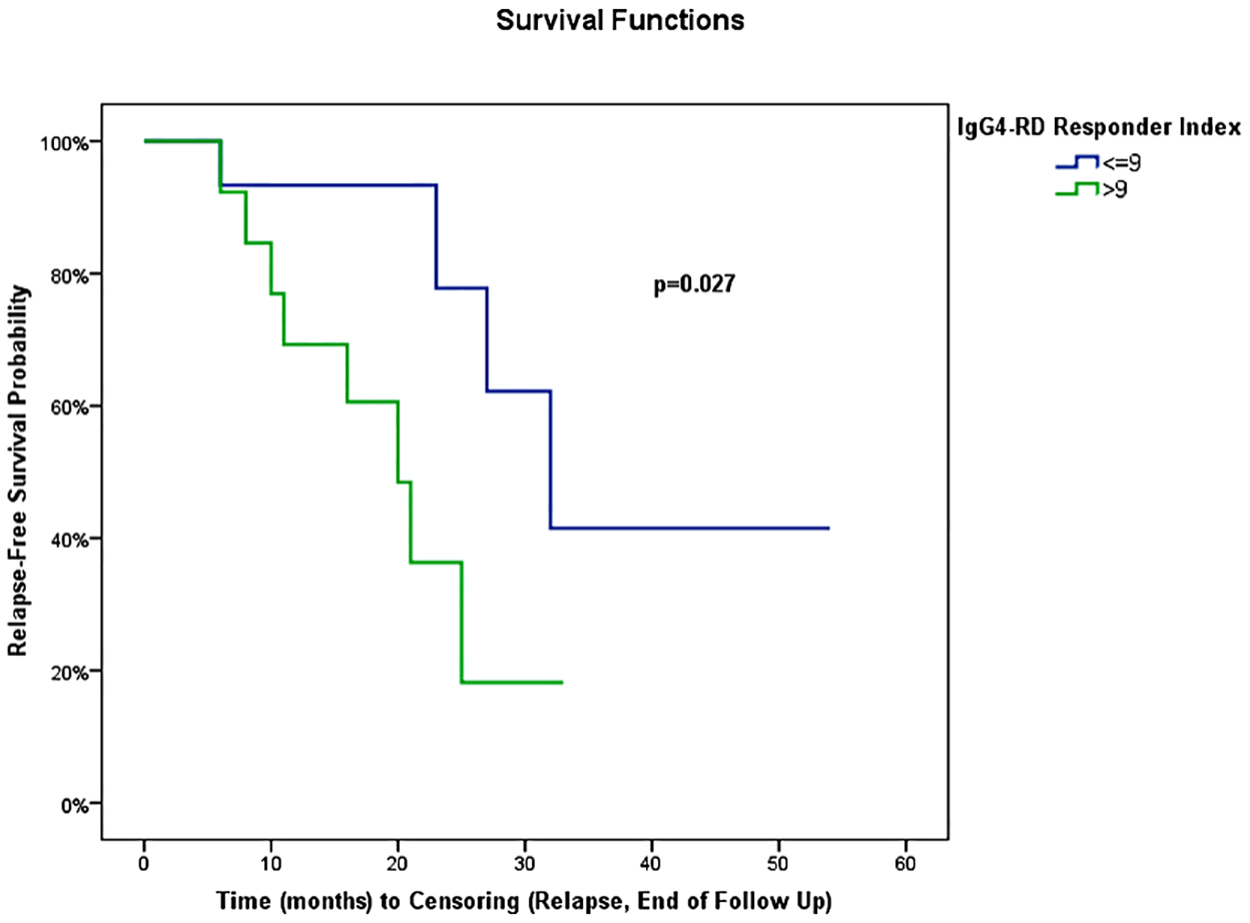
Kaplan-Meier plots of the risk of disease relapse following rituximab for patients with IgG4-RD RI >9 and with IgG4-RD RI ≤9 at RTX treatment. IgG4-RD RI, IgG4-related disease Responder Index.

**Fig 3:**
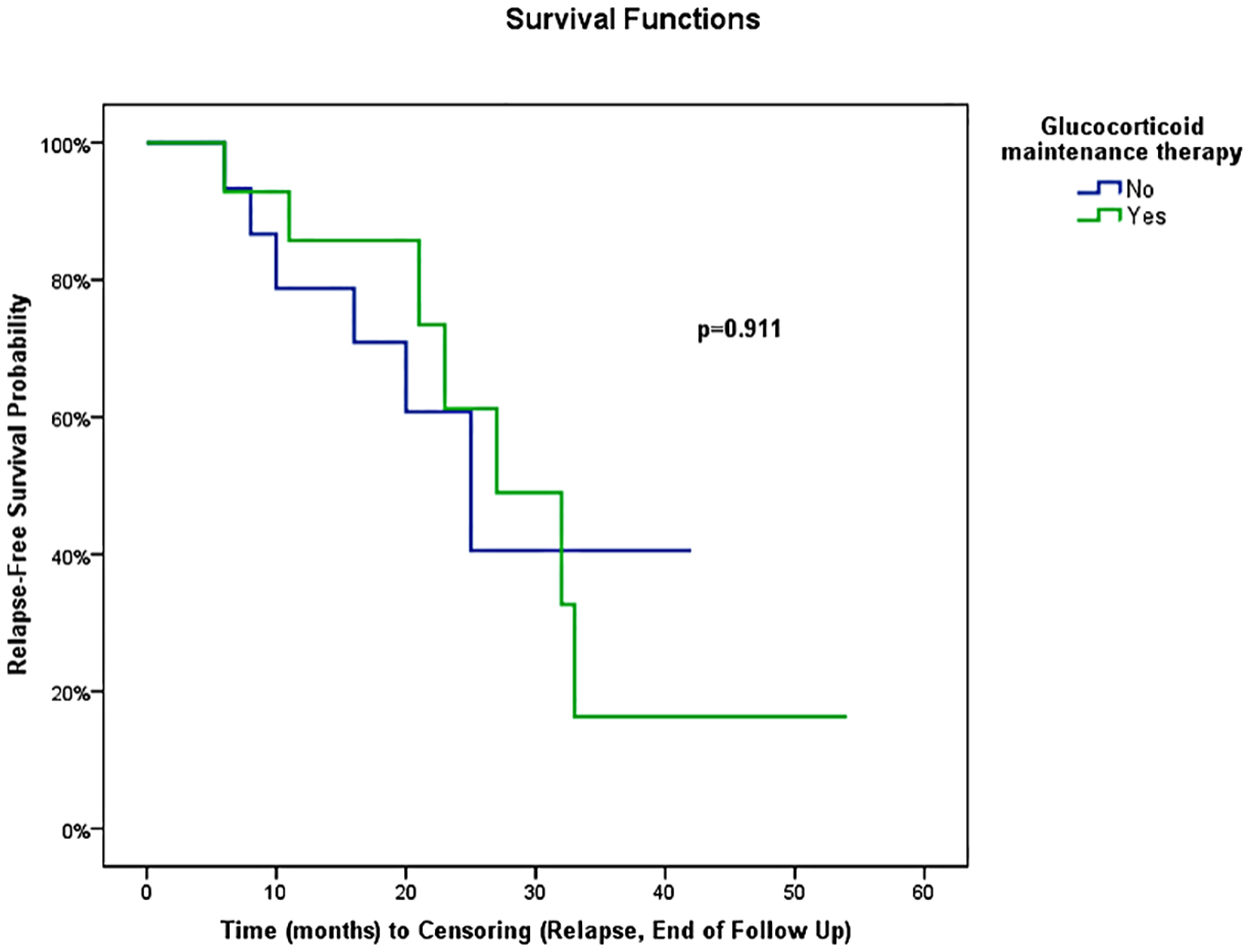
Risk of disease relapse following rituximab for patients with glucocorticoid and without glucocorticoid maintenance therapy at last follow-up or relapse.

**Fig 4:**
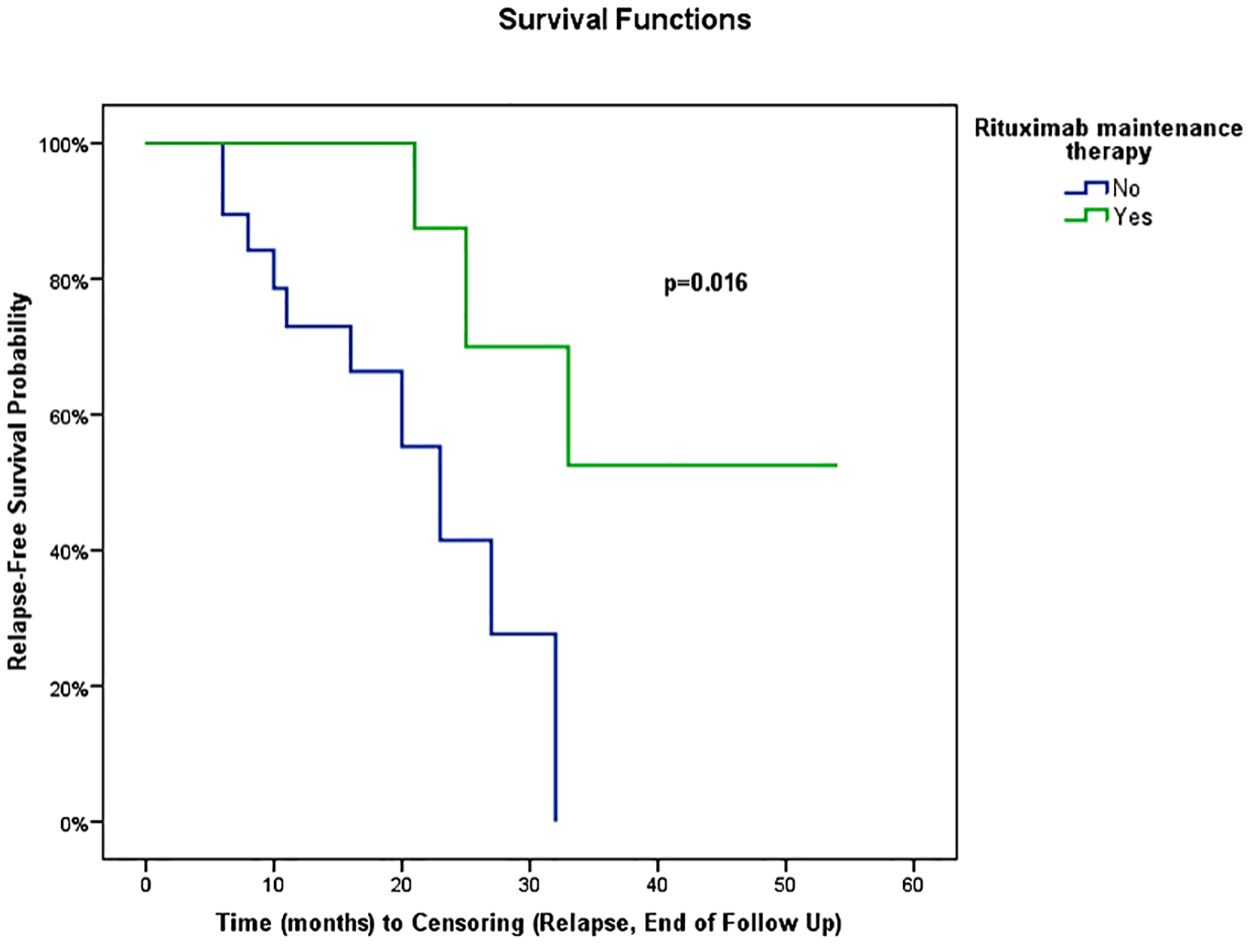
Risk of disease relapse for patients with or without “prophylactic” maintenance therapy with rituximab.

In univariate analysis using Cox regression, gender, age at RTX onset, disease duration before RTX treatment (<18 versus ≥18 months), number of involved organs (<3 versus ≥3) or type of involved organ (kidney, pancreas, bile ducts, or retroperitoneum) were not associated with the occurrence of relapse (Table 4). IgG4-RD RI >9 at the time of RTX onset was the only variable associated with relapse (HR = 3.68, 95% CI: 1.07, 12.62) (*P* = 0.04) (Table 4). When IgG4-RD RI was >9, mean relapse-free survival was 20 ±3 months (95% CI: 14.4, 24.8) versus 37 ±6 months (95% CI: 25.8, 48.6) in patients with IgG4-RD RI ≤9 (*P* = 0.02) (Fig 2). Biological variables analyzed were not statistically associated with relapse using Cox regression (Table 4). The type of RTX regimen (1 g d1-d15 regimen versus 4 weekly 375 mg/m^2^ regimen, *P* = 0.27), the number of treatment lines before RTX (≥2 or <2, *P* = 0.56), or the maintenance of long-term GC therapy at last follow-up or relapse (*P* = 0.91, Fig 3) were not statistically associated with relapse. Systematic (i.e. before relapse) RTX maintenance retreatment was statistically associated with a longer relapse-free survival (mean relapse-free survival: 41.1 ±5.6 months, 95% CI: 30.2, 52.1) compared with patients who were not systematically retreated (mean relapse-free survival: 21.3 ±2.5 months, 95% CI: 16.3, 26.2) (*P* = 0.02, Fig 4). In a bivariate model, IgG4-RD RI >9 and systematic RTX maintenance retreatment were independently and statistically associated with relapse (IgG4-RD RI >9 was predictive of relapse with HR = 7.64; 95% CI: 1.44, 40.43; *P* = 0.02, while systematic RTX maintenance retreatment was protective of relapse with HR = 0.10; 95% CI: 0.02, 0.69; *P* = 0.02).

**Table 4:** Hazard ratio for risk of flare: Unadjusted Cox regressions.

Variable	Hazard ratio	95% CI	*P*-value
Demographics			
Male, gender	1.49	0.33, 6.76	0.605
Age at RTX, continuous	0.97	0.92, 1.02	0.234
Clinical-radiological variables			
Number of involved organs (≥3 organs)	2.23	0.61, 8.14	0.225
Kidney involvement	0.64	0.20, 2.03	0.450
Pancreas involvement	1.08	0.36, 3.29	0.887
Bile ducts involvement	0.72	0.23, 2.27	0.577
Retroperitoneum involvement	1.31	0.28, 6.20	0.733
**IgG4-RD RI (>9)**	**3.68**	**1.07, 12.62**	**0.038**
Biological variables before RTX			
Serum IgG4 (≥135 mg/dl)	1.38	0.30, 6.40	0.685
Hypergammaglobulinemia (>15 g/l)	1.10	0.37, 3.29	0.864
Eosinophilia (>0.5 G/l)	0.60	0.16, 2.24	0.450
Hypocomplementemia	1.31	0.32, 5.29	0.705
CRP (>10 mg/l)	1.03	0.31, 3.50	0.957
Treatment variables			
Therapeutic lines before RTX (≥2)	1.4	0.45, 4.48	0.559
RTX regimen (1 g x2 vs 375 mg/m^2^ x4)	2.39	0.50, 11.37	0.273
GC maintenance therapy	0.94	0.31, 2.83	0.911
**RTX prophylactic maintenance therapy**	**0.18**	**0.04, 0.84**	**0.029**

Values in bold show statistical significance. CI, confidence interval; CRP, C-reactive protein; GC, glucocorticoid; RI, Responder Index; RTX, rituximab.

### RTX retreatment

Seventeen (51.5%) patients received more than one course of RTX, with a total number of 58 retreatment courses. The median number of retreatment per patient was two (range: 1–12). Retreatment was used as systematic in 12 cases, and for relapses in 9.

Systematic retreatment (maintenance) RTX therapy was performed in the context of orbital disease (n = 4), IgG4-related sclerosing cholangitis (n = 3), IgG4-related tubulointerstitial nephritis (n = 3), and IgG4-related retroperitoneal fibrosis (n = 2). Doses for maintenance were variable ranging from 300 mg to 1 g, every month to 17 months. Relapse after systematic RTX retreatment occurred in 4/12 (33%), with a median delay of 17 months after the last RTX infusion (range: 14–18). Follow-up since last maintenance RTX infusion was shorter than 12 months in 7/8 patients otherwise still in remission.

All patients but one that had been retreated for relapses had initially responded to the first course of RTX. The non-responder patient did not respond to a second course of RTX. A clinical response was obtained in all seven other evaluable patients retreated for relapse.

### B-cell depletion and disease relapse

B-cell lymphocyte counts in the first year following RTX treatment were available in 24 patients. Complete B-cell depletion (CD19+ B-cells = 0) and partial depletion (CD19+ B-cells ≥1 but <10/mm^3^) were obtained in 17 (70.8%) and 5 (20.8%) patients, respectively. Two patients (8.3%) had >10/mm^3^ CD19+ B-cells despite treatment. Among the 14 patients with initial B-cell complete or partial depletion and longitudinal follow-up available, a reconstitution of B-cell compartment (CD19+ B-cells >10/mm^3^) was observed in 8 (57.1%) patients, with a median delay of 12.5 months (range: 2–29) after the last RTX infusion. Six (75%) of these 8 patients with B-cell reconstitution presented a clinical relapse, compared with only 2/6 (33.3%) of patients without B-cell reconstitution.

### Safety / Tolerance

No deaths were observed during follow-up.

From a total of 140 RTX infusions, a hypersensitivity reaction within the 24 hour post-infusion period was observed in 2 cases: 1 case each of palpebral and pharyngeal edema (1 event/70 perfusions).

Eighteen infectious episodes were reported in 11 (33.3%) patients. Eight infections were considered as severe (requiring intravenous antibiotherapy or hospitalization) and occurred in 4 patients: 1 patient each with recurrent urinary and biliary sepsis with Gram-negative and *Staphylococcus aureus* bacteremia, recurrent anal abscesses, *Staphylococcus hominis* mitral endocarditis, and recurrent angiocholitis with Gram-negative bacteremia during biliary relapses (rate of severe infections of 12.1/100 patient-years). The 10 other infections (especially ear, nose and throat or low urinary tract infections) were mild and occurred in 7 patients.

No case of RTX-related neutropenia was observed. Gammaglobulin level was <7 g/L during follow-up in 5 (15.1%) patients, and hypogammaglobulinemia (Ig ≤5 g/L) was observed in 3 patients. One of them, treated every 3 months with RTX infusions for severe IgG4-related cholangitis, developed profound hypogammaglobulinemia in the first year of treatment and ultimately required intravenous immunoglobulin (IVIg) substitution.

## Discussion

Advances in the management of IgG4-RD requires cohort studies to identify clinical subgroups and risk factors for relapses that will help clinicians to determine the optimal treatment strategy in order to prevent disease flares [27]. Herein, we report on the use of B-cell depletion with RTX in 33 patients with IgG4-RD. A major strength of this retrospective study is the long term follow-up after the first RTX infusion (mean follow-up > 2 years). As previously reported, our data confirm that RTX is highly effective in inducing disease remission of IgG4-RD, with 93.5% of patients presenting a clinical response after onset of therapy. Yet, we also observed a high rate of relapse (42%) which was associated in univariate analysis with high baseline disease activity (as defined by IgG4-RD RI (excluding serum IgG4 levels) >9). Of note, all patients who initially responded to RTX but ultimately relapsed were successfully challenged with a second course of RTX. In addition, the use of a “maintenance” therapy by systematic RTX infusions was associated with an increase of relapse-free survival, with however a probably temporary effect.

Demographic and clinical characteristics from IgG4-RD patients of the present study are in line with data from previous cohorts [28–32]. Most patients (57.6%) presented with a systemic form of the disease (i.e. ≥3 involved organs), whereas localized disease was otherwise found in 30.3%. Assessment of disease activity by IgG4-RD RI [26], without serum IgG4 because of growing knowledge that serum IgG4 level has shortcomings as a disease biomarker [20,33,34], revealed active disease before RTX treatment with a mean IgG4-RD RI of 10 ±6.1. Clinical, biological, and radiological evaluations were used separately to analyze response to treatment rather than IgG4-RD RI because of the retrospective and multicenter character of the study. Indeed, the rate of follow-up visits and the means of radiological evaluation varied among patients, which did not allow to accurately and homogeneously score patients with the IgG4-RD RI during follow-up. Clinical response rate observed after RTX in our study was high (93.5%), as previously described [17,18,20]. The only two non-responders presented with fibrotic lesions (maxillary IPT and pachymeningitis) that could be an explanation to the lower effect of B-cell depletion [17,35]. Besides this high rate of clinical improvement, radiological normalization was obtained in less than one third of patients with conventional radiologic procedures (CT or MRI) and less than one half of patients by ^18^FDG PET/CT. Persistent abnormalities in conventional imaging could in part be explained by “scar-fibrotic” lesions. Yet, persistent hypermetabolisms in ^18^FDG PET/CT imaging rather suggests relentless active inflammation, not fully curbed by B-cell depletion.

In our study, with a mean follow-up of 25 months, the relapse rate after treatment with RTX was 42%. The short follow-up available in previous studies makes the comparison difficult [17,20]. Nevertheless, in a recent retrospective US cohort study of 60 patients with IgG4-RD treated with RTX, a relapse rate of 37% was reported in responders [18], with a median delay of relapse of 244 days (8 months), which is shorter than in our study (17 months). The concomitant use of GC in the majority of our patients and maintenance of DMARDs in 4 cases with RTX as add-on therapy is a possible explanation for this difference. Indeed, more than two-thirds of the patients in the US study were treated without concomitant GC, except for methylprednisolone premedication [18]. However, the maintenance of a long-term GC therapy after RTX treatment was not associated with a longer relapse-free survival in our study, on the contrary to the results of a recent randomised controlled trial in patients with autoimmune pancreatitis [36].

Neither older age or male sex (unlike previously reported [37]), nor disease duration before RTX, number of involved organs or type of organ involvement were associated with the occurrence of relapse by Cox regression analysis in this study. Conversely, a high activity of the disease, as defined by an IgG4-RD RI >9, was associated with a shorter relapse-free survival after RTX treatment (20 versus 37 months), whereas baseline serum Ig4 and eosinophils were not associated with relapse, on the contrary to the results of the Massachusetts General Hospital experience [18].

Few data are available regarding retreatment with RTX in IgG4-RD. This study shows that systematic maintenance retreatment with RTX was associated with a longer relapse-free survival (41 months versus 21 months, *P* = 0.02). A total of 17 patients received more than one course of RTX in this study, including systematically retreated patients and patients retreated for relapse. In the study by Wallace and colleagues, response to retreatment was not included in the relapse-free survival analysis because censoring time included relapse, end of follow-up, and also retreatment [18]. In the prospective, open-label trial by Carruthers and colleagues, six patients had previously been treated with RTX before study inclusion, and four patients were retreated for relapse during the 12 months after enrolment [20]. However, response or tolerance of retreatment were not specified. Finally, Yamamoto and colleagues recently described three cases sequentially retreated with 1 to 6 courses of RTX over a period of 4 years, and reported in a patient an attenuation of RTX’s efficacy over time [21]. This tendency was not observed in our patients. However time to relapse after systematic maintenance retreatment appeared to be similar to time to relapse in the whole population treated by RTX (median delay of 17 months), suggesting an only temporary effect of this systematic retreatment. Finally, dosage, frequency and duration of infusions in such strategy must be determined in the context of IgG4-RD, as has been done in other indications, such as ANCA-associated vasculitis (AAV) [38]. In the same way, since relapses are frequent after B-cell reconstitution and appear to be rare without CD19 B-cell detection, individually tailored RTX-infusion schedules based on CD19 B-cell reappearance, as recently reported during AAV [39], should be further studied in IgG4-RD.

The high rate of relapse observed after RTX treatment in our study also raises pathophysiological issues. Indeed, as described in recent studies, immunological changes during IgG4-RD are not restricted to B-cell compartment but also involve T cells, such as CD4+ cytotoxic T lymphocytes [40,41] or T follicular helper cells [42–44]. These possibly causal T-cell dysfunctions could account for the *de novo* oligoclonal expansion of circulating plasmablasts observed after RTX treatment [33], and relapses observed in a number of our patients. Plasmablast counts were not used in clinical practice in our study, but a relapse was observed in 75% of patients with CD19+ B-cell reconstitution. The observation of a relapse in 2 patients without circulating B-cell reconstitution is another argument suggesting a potential role of T cells in the disease’s pathophysiology, and the rational to target these cells in future studies, as recently reported with abatacept [45].

Finally, the number of RTX infusions analyzed in 33 patients in this study, along with the relatively long follow-up available in most patients (27 and 10 patients with ≥12 months and ≥24 months of follow-up, respectively), also provides important data regarding the tolerance of RTX in IgG4-RD. Hypersensitivity reactions following RTX infusions (possibly mediated by a rheumatoid factor in IgG4-RD in a recent report [46]), appeared to be rare in our series (1 event/70 perfusions). However, the rate of severe infections observed in our study (12.1/100 patient-years) was indeed relatively high when compared to the use of RTX in other autoimmune diseases (5.0/100 patient-years in rheumatoid arthritis [47], 6.6/100 patient-years in systemic lupus erythematosus [48], and 2.3/100 patient-years in immune thrombocytopenia [49]) and similar to the rate of infections observed in non-viral cryoglobulinemia vasculitis (14.1/100 patient-years) [50]. The eventual role of concomitant or previous treatments, including corticosteroids, could in part explain this high risk of severe infections. However, in the four patients with severe infections, none received DMARDs before or after RTX treatment, and exposure to steroids before RTX was <1 year (1, 2, 5 and 11 months). Only one of these four patients presented with hypogammaglobulinemia (requiring IVIg substitution). Hypogammaglobulinemia was found in 4 other patients (n = 5, 15.1%), ≤5 g/L in 3 patients in our study, which is a higher rate than that observed in other autoimmune conditions [49]. Other potential reasons for this high rate of severe infections could be related to intrinsic factors of the disease. First, compressive complications occurring in biliary or urinary tracts could account for a specific risk of obstructive infectious events. Next, specific biases related to the design of the study (e.g. the small sample size and selection bias) could represent additional explanations. Yet, although expansion of peripheral blood lymphocyte subsets, such as plasmablasts and CD4^+^ cytotoxic T lymphocytes, have been described in IgG4-RD patients [33, 34, 40], no decrease of immune cell subsets or immune-deficient state has yet been associated with IgG4-RD, including in patients treated with RTX so far. Hence, until a prospective randomized-controlled trial be performed in this specific condition, and given the large experience built with rituximab in other conditions, we believe that the present data should not be a hindrance to this therapeutic approach in IgG4-RD.

There are a number of limitations to our study. First, its retrospective design could cause bias in data collection. The multicenter and retrospective nature of the study could also explain heterogeneity in evaluation of treatment response, especially in modalities and frequency of radiological evaluations. Next, the limited number of patients and relapses studied here could represent a limitation of power to identify potential risk factors of relapse in these patients. Finally, the use of concomitant treatments could also confound the evaluation of response, relapse and tolerance following RTX treatment. Hence, drawing conclusions regarding the efficacy and safety of RTX in this context should be made with caution.

In conclusion, this retrospective multicenter study suggests that RTX is an effective treatment for both remission induction and treatment of relapses in IgG4-RD. Nevertheless the relapse rate remains high after treatment (approximately half of the patients reported in the present series) and patients with the most active disease appear to be at higher risk of relapse. Systematic RTX retreatment could represent a therapeutic strategy to prevent relapse. Yet, considering the high rate of severe infections and occurrence of hypogammaglobulinemia in some patients, such a strategy should be restricted to patients with IgG4-RD RI >9 before treatment. Despite the rarity of IgG4-RD and the challenge met by the recruitment of patients, randomized controlled trials are eagerly awaited in this condition, in order to compare rituximab with other steroid-sparing treatments. Last, the temporary effect of this treatment and recent data concerning the role of T cells in the pathophysiology of the disease should prompt clinicians to evaluate other targeted treatments in IgG4-RD.

## “Take-home” messages / Highlights

- Rituximab (RTX) is effective for induction of remission in IgG4-related disease.
- Up to 40% of RTX-treated patients relapse after 2 years of follow-up.
- Baseline high disease activity (IgG4-RD RI>9) and the lack of maintenance therapy with RTX are risk factors of relapse.
- Hypogammaglobulinemia and infections are reported in a few patients treated with RTX.

## Supporting information

S1 Fig Flow chart of the “French rituximab—IgG4-RD study”. (TIF)
