# Supplementary figures and images for: Long-term efficacy and safety of rituximab in IgG4-related disease: Data from a French nationwide study of thirty-three patients
Source: PLoS One. 2017 Sep 15;12(9):e0183844. doi: 10.1371/journal.pone.0183844 (PMC5600376; doi:10.1371/journal.pone.0183844)

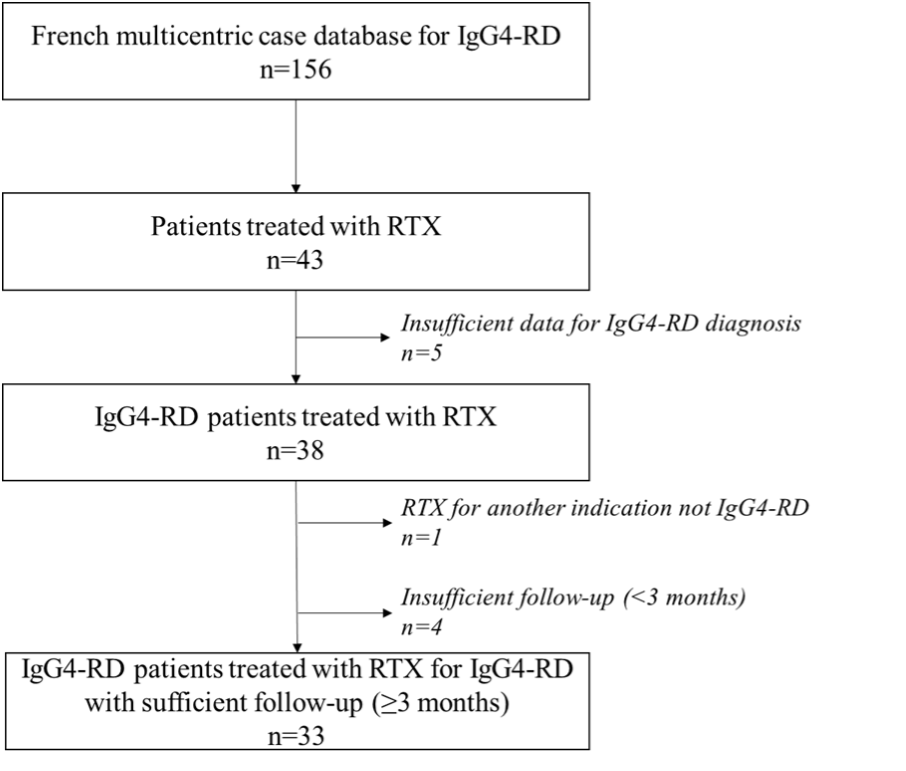

Supplement: S1 Fig — (TIF) [file pone.0183844.s001.tif]
